# Benzo[*g*]coumarin-Based Fluorescent Probes for Bioimaging Applications

**DOI:** 10.1155/2018/5249765

**Published:** 2018-06-14

**Authors:** Yuna Jung, Junyang Jung, Youngbuhm Huh, Dokyoung Kim

**Affiliations:** ^1^Department of Biomedical Science, Graduate School, Kyung Hee University, 26 Kyungheedae-Ro, Dongdaemun-Gu, Seoul 02447, Republic of Korea; ^2^Department of Anatomy and Neurobiology, College of Medicine, Kyung Hee University, 26 Kyungheedae-Ro, Dongdaemun-Gu, Seoul 02447, Republic of Korea; ^3^Center for Converging Humanities, Kyung Hee University, 26 Kyungheedae-Ro, Dongdaemun-Gu, Seoul 02447, Republic of Korea

## Abstract

Benzo[*g*]coumarins, which consist of coumarins fused with other aromatic units in the linear shape, have recently emerged as an interesting fluorophore in the bioimaging research. The pi-extended skeleton with the presence of electron-donating and electron-withdrawing substituents from the parent coumarins changes the basic photophysical parameters such as absorption and fluorescence emission significantly. Most of the benzo[*g*]coumarin analogues show red/far-red fluorescence emission with high two-photon absorbing property that can be applicable for the two-photon microscopy (TPM) imaging. In this review, we summarized the recently developed benzo[*g*]coumarin analogues including photophysical properties, synthesis, and applications for molecular probes that can sense biologically important species such as metal ions, cell organs, reactive species, and disease biomarkers.

## 1. Introduction

Coumarin (2*H*-chromen-2-one) is a chemical compound in the benzopyrone chemical class that can be found in many natural species [1, 2]. Coumarins possess variety of biological activities and unique photophysical properties. Among them, the fluorescent property received much attention recently due to its high quantum yield, high stability, and biological compatibility [3, 4]. The coumarin-based fluorescent dyes and molecular probes have been applied not only for basic science such as physics, chemistry, medical science, and clinical science but also for industry and engineering [5, 6]. In progress, various kinds of the expanded or combined structure of coumarin derivatives have been discovered. Among them, linearly extended derivatives, benzo[*g*]coumarin, show superior photophysical properties in the bioimaging applications over the other derivatives [7]. Moreover, a large two-photon absorbing ability with longer excitation and emission wavelengths in optical window, high photostability, and high chemical stability are the key advantageous features of benzo[*g*]coumarin derivatives. In this review, we describe a brief explanation of benzocoumarin analogues with photophysical properties, their synthetic methods, and the recently developed benzo[*g*]coumarin-based one/two-photon excitable fluorescent dyes/probes that can sense biologically important species.

## 2. Benzo[*g*]coumarins

**Figure 1:**
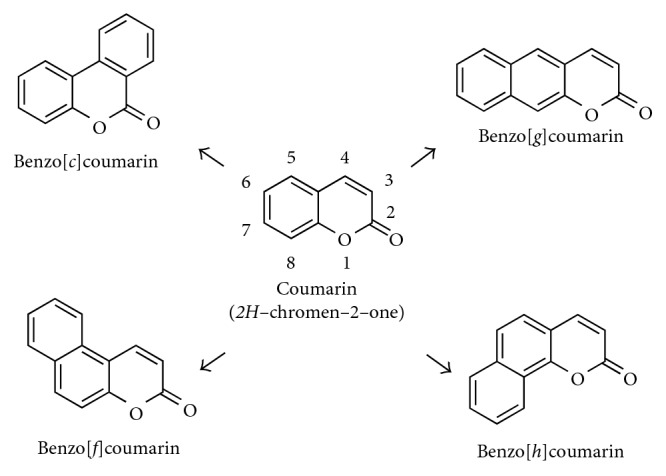
Chemical structures of coumarin and benzocoumarin derivatives.

Benzocoumarin derivatives can be categorized into four types depending on the position of the fused aromatic ring in the parent coumarin backbone (Figure 1): (1) benzo[*c*]coumarin (3,4-benzocoumarin) fused on 3,4-position; (2) benzo[*g*]coumarin (6,7-benzocoumarin) fused on 6,7-position; (3) benzo[*f*]coumarin (5,6-benzocoumarin) fused on 5,6-position; and (4) benzo[*h*]coumarin (7,8-benzocoumarin) fused on 7,8-position.

### 2.1. Photophysical Properties of Benzo[*g*]coumarin Analogues

**Figure 2:**
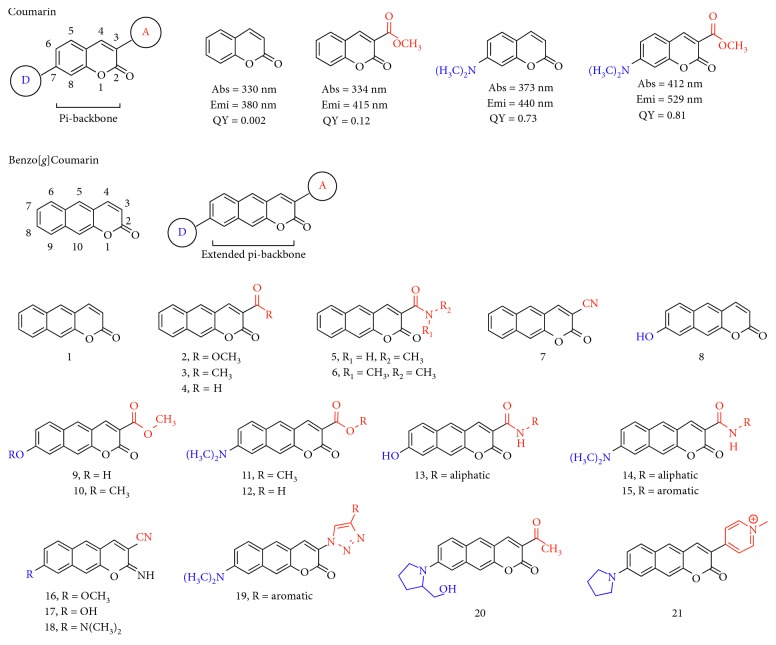
Chemical structure and basic photophysical properties of coumarin and benzo[*g*]coumarin derivatives. D: electron-donating group; A: electron-accepting group. The wavelengths are derived from the highest intensity values in the absorption and fluorescence emission spectra.

The photophysical property such as absorption and fluorescence emission of coumarin derivatives has been identified by many scientists in various fields. Among the derivatives, the functional group substitution on the 3- and 7-position gives large property changes from the original backbone. Typically, coumarin itself shows maximum absorbance and emission at a short wavelength (*λ*_max,abs_ = 330 nm; *λ*_max,emi_ = 380 nm) with poor fluorescence quantum yield (Φ_F_) (Figure 2) [7]. However, the appropriate substitution, electron donor-acceptor- (D-A-) type structure on the pi-backbone, induces intramolecular charge transfer (ICT) accompanied with the quantum yield increment and the red shift of the fluorescence emission wavelength (see the absorption and emission wavelengths with quantum yield information in Figure 2) [8, 9].

Considering the bioimaging application of the coumarin fluorophore, the excitation and emission at a shorter wavelength give drawbacks such as (i) interference of autofluorescence from the biological substances, (ii) limited imaging depth, (iii) photodamage of the sample, and (iv) photobleaching of the fluorophore [10, 11]. In that sense, pi-extended benzo[*g*/*f*/*h*]coumarins are expected to provide several advantageous features over the corresponding coumarins due to their extended aromatic backbone that evokes longer wavelength absorption and fluorescence emission. Also, the conformationally restricted pi-backbone extension gives high photostability with a high quantum yield.

The photophysical properties of benzocoumarin analogues may also be predicted based on the type and substitution position. Recently, Ahn et al. reported a systematic analysis result for photophysical properties of benzocoumarin analogues [12]. They revealed that linear-type benzo[*g*]coumarins give similar substitution-induced property changes like parent coumarins, and the fluorescence property is suitable for bioimaging application over the others (very poor or no fluorescence from the benzo[*f*/*h*]coumarin in aqueous media).

The electron-withdrawing functional group-substituted benzo[*g*]coumarin at 3-position induces a red shift of fluorescence from the parent benzocoumarin (**1**, *λ*_max,emi_ = 459 nm); ester moiety (**2**, 534 nm), ketone (**3**, 549 nm), aldehyde (**4**, 547 nm), amide (**5**, 515 nm), and nitrile (**7**, 533 nm) (Figure 2; Table 1). Interestingly, the combination of electron-withdrawing and electron-donating moieties at 3- and 8-position gives a significant shift of both absorption and emission spectra to the longer wavelength region (**8**–**21**; Figure 2; Table 1). The substitution of the hydroxyl group alone at 7-position (electron-donating position) shows no significant red shift (*λ*_max,emi_ = 466 nm) (**8**), but the combination with electron-withdrawing substitution (methyl ester) at 3-position induces large changes (**9**, **10**, and **13**; *λ*_max,emi_ > 500 nm). The alkylamine (-NR_2_, R = alkyl) substitution at 8-position gives more significantly red-shifted absorption (>430 nm) and fluorescence emission (>580 nm) with a combination of ester, amide, nitrile, triazole, and pyridinium salts (**11**–**21**). The details of each compound with their photophysical properties are covered in the next chapter with the reported applications.

### 2.2. Synthesis of Benzo[*g*]coumarin Analogues

**Figure 3:**
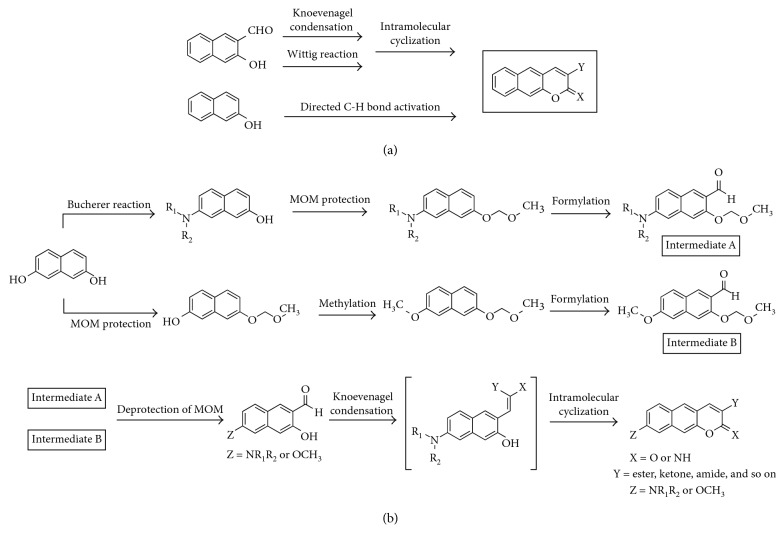
Synthetic routes for benzo[*g*]coumarin derivatives. (a) Routes for nonsubstitution at C-8 position. (b) Routes for substitution at C-8 position. X = O or NH; Y = ester, ketone, amide, nitrile, and so on; Z = NR_1_R_2_ or OR (R = alkyl).

Synthesis of benzo[*g*]coumarin analogues follows the established synthetic routes including the Knoevenagel condensation or Wittig reaction with intramolecular cyclization reaction to *o*-hydroxynaphthaldehyde (Figure 3(a)) [7]. Alternatively, the direct electrophilic substitution of naphthols with *β*-keto ester followed by cyclization also gives benzo[*g*]coumarin in the presence of a catalyst. The metal-catalyzed aryl C–H functionalization of alkynoates is also feasible (Figure 3(a)).

Synthetic methods to make an electron-donating moiety on benzo[*g*]coumarin analogues were developed by many scientists including Ahn et al. [12, 15]. Representative derivatives which have a primary/secondary amine or a hydroxy/methoxy moiety at the 8-position were synthesized from the key intermediates A and B (Figure 3(b)). Intermediate A analogues were prepared by monoamination through the Bucherer reaction, protection of the hydroxyl group by methoxymethyl ether (MOM), and formylation through directed lithiation. On the contrary, methoxy or hydroxy group-substituted intermediate B analogues were prepared by monoprotection of the hydroxyl group by MOM first and then by methylation and formylation [12].

The MOM deprotection of the intermediates A and B in acidic condition gives an *o*-hydroxynaphthaldehyde intermediate, and the cascade intramolecular cyclization reaction generates benzo[*g*]coumarin derivatives (Figure 3(b)) [15, 19].

## 3. Benzo[*g*]coumarins for Fluorescent Probes

Benzo[*g*]coumarin derivatives have been used in various research areas. In particular, their unique photophysical property gives many advantages in the bioimaging applications such as fluorescent probes and tags and photolabile materials.

Recently, a few examples of notable applications using benzo[*g*]coumarin derivatives for the fluorescent probes were reported. The fluorescent probe is undoubtedly an essential and useful tool in the biological, medical, and environmental sciences to investigate molecular interactions and biological activities, among others [23]. As we described above, benzo[*g*]coumarin analogues with suitable substitution show the absorption and fluorescence emission at the longer wavelength region (red and near-infrared) that gives better cellular or tissue imaging results than the shorter wavelength. Moreover, the pi-extended structure with proper substitutions gives a sufficiently large two-photon absorbing property that is applicable for the two-photon excitation microscopy [19]. Two-photon excitation microscopy is a fluorescence imaging technique that allows imaging of ex vivo and *in vivo* tissue up to millimeter depths [10, 24].

In this chapter, we summarized recently reported fluorescent probes based on the benzo[*g*]coumarin analogues (one/two-photon absorbing) with their interesting applications in the (i) sensing and bioimaging of biologically important species including metal ions, cell organs, reactive oxygen species (ROS), and disease biomarkers and (ii) deep tissue imaging.

### 3.1. Sensing of Metal Ions

#### 3.1.1. Copper Ions (Cu^2+^)

Copper ion plays crucial roles in living systems including signal transduction, oxygen transportation via copper metalloenzymes, cellular energy generation, and cofactors of protein activity. As a result, the homeostasis of copper ions in the biological system is very important and directly related with various diseases: Alzheimer's disease (AD), Wilson's disease, Prion disease, and Menkes disease [25, 26].

**Figure 4:**
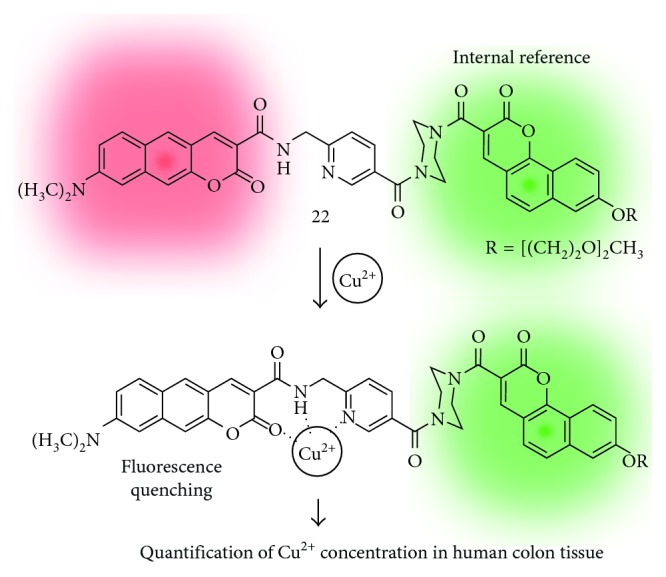
Benzo[*g*]coumarin- and benzo[*h*]coumarin-based fluorescent probe for the copper ion (**22**).

Cho et al. reported a fluorescent probe for Cu^2+^ and quantitatively estimated ion concentrations in human tissues by two-photon microscopy imaging and analysis [27]. The amide- and dimethylamino-substituted benzo[*g*]coumarin analogue is linked with a benzo[*h*]coumarin analogue as an internal reference (internal reference: insensitive toward substrates or environment and maintains a steady fluorescence intensity) (**22**; Figure 4). The fluorescence intensity of benzo[*g*]coumarin at the red region (emission: 550–650 nm) was decreased through a chelation to the copper ion with a piperazine linker. Benzo[*g*]coumarin analogue (sensing part) and benzo[*h*]coumarin analogue (internal reference part) serve sufficiently high two-photon action cross section (Φ*δ*, GM value), 32 GM and 46 GM in ethanolic water (EtOH/HEPES 9 : 1 v/v, pH 7.0) at 750 nm two-photon excitation, respectively. Probe **22** shows high sensitivity (0.84 *μ*M) and selectivity toward Cu^2+^ with no perturbation due to high concentration of biological alkali and alkaline earth metal ions. They investigated the quantitative estimation of the Cu^2+^ concentration in live cells, rat brain tissue, and human colon tissue samples by using two-photon microscopy and analyzed the results. The higher concentration of Cu^2+^ in the cancer tissue (22 ± 3 *μ*M) was observed than in polyp (13 ± 2 *μ*M) or normal (8.2 ± 0.3 *μ*M) samples, and it revealed that estimation of Cu^2+^ concentration may be useful for the diagnosis of colon cancer.

#### 3.1.2. Sodium Ions (Na^+^)

**Figure 5:**
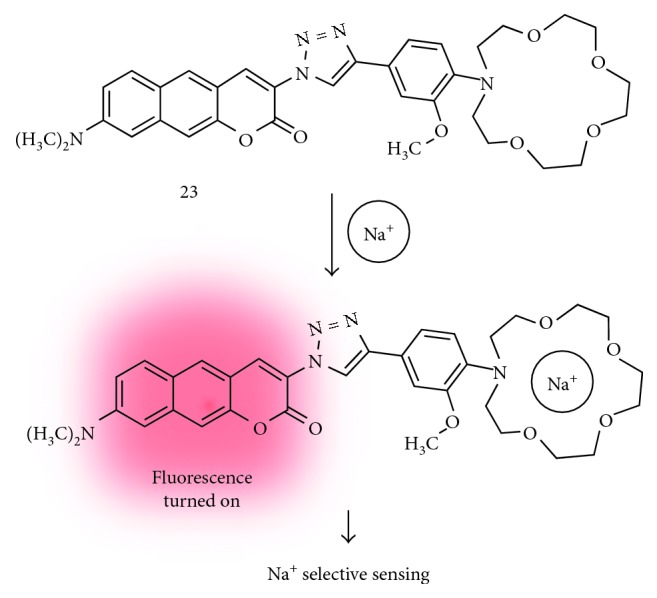
Benzo[*g*]coumarin-based fluorescent probe for the sodium ion (**23**).

Sodium ion is one of the most important analytes in life science. It is necessary for live species for the nerve impulses, heart activity, metabolic functions, and biological balance [28, 29]. The Na^+^ concentration range in the intracellular (5–30 mM) and extracellular (100–150 mM) space was observed, and competitive cation K^+^ also showed similar concentrations. Accordingly, a development of tools for selective detection of Na^+^ over K^+^ is very important and challenging. Recently, Holdt et al. designed a Na^+^ selective fluorescent probe based on the benzo[*g*]coumarin derivatives which have an *N*-(*o*-methoxyphenyl)aza-15-crown-5 moiety (**23**; Figure 5) [20]. A higher Na^+^/K^+^ selectivity of **23** was observed, but also, it gives a higher *K*_d_ value (223 mM) as a limitation for detection of lower concentration of Na^+^. In this study, the bioimaging application was not reported, but they proposed a design strategy to develop benzocoumarin-based fluorescent probes.

#### 3.1.3. Mercury Ions (Hg^2+^)

**Figure 6:**
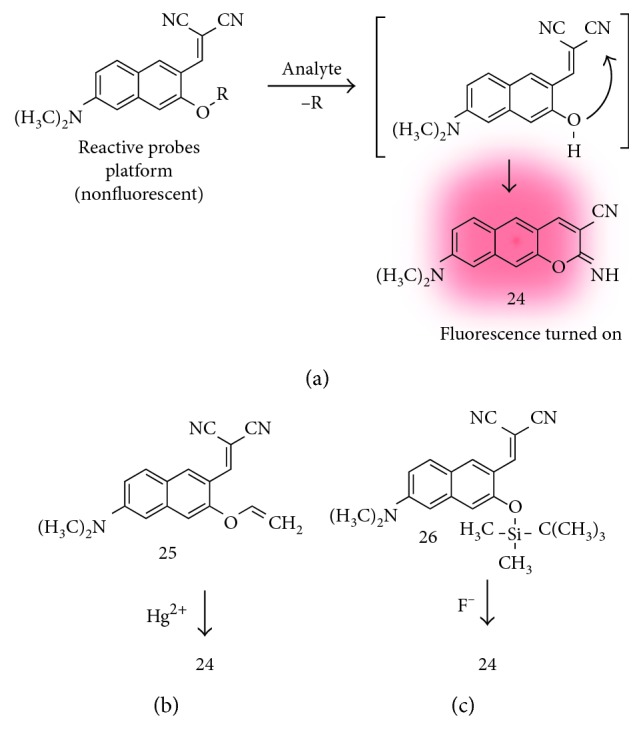
(a) Iminobenzo[*g*]coumarin analogue (**24**) and structure of its precursor (reactive probes platform) with a proposed sensing mechanism. Reaction-based fluorescent probes for mercury ion (**25**) (b) and fluoride ion (**26**) (c).

Mercury ion is a chemical that is widely used in industry and basic science [30]. However, mercury is a highly poisonous element and causes damage to the central nervous system and other organs. So far, various kinds of detecting methods for mercury species (Hg^2+^, MeHg^+^, etc.) including fluorescent probes have been developed [30]. By using the latent probe approach with the benzo[*g*]coumarin platform, Ahn et al. reported new fluorescent probes for mercury ion sensing (Figure 6) [15]. The cleavage of sensing moiety on the platform by selective and sensitive chemical reaction toward the target analyte generates a chemically unstable intermediate, which undergoes a fast cyclization reaction to afford an iminobenzo[*g*]coumarin derivative (**24**; Figure 6(a)). For the mercury ion sensing, they introduce a vinyl ether group to the Hg^2+^-promoted hydrolysis (**25**; Figure 6(b)). The probe shows negligible fluorescence emission in the aqueous media due to the generation of the free rotation-induced nonradiative decay pathway from the dicyanoalkene moiety (molecular rotor moiety) and gives significant fluorescence enhancement upon adding mercury ions followed by iminobenzo[*g*]coumarin formation. Compound **24** shows absorption and fluorescence emission maximum at 446 nm and 585 nm, respectively, with a high quantum yield (QY = 0.67).

#### 3.1.4. Fluoride Ions (F^−^)

Fluoride ion plays an important role in chemistry, environment, medicine, and biology. Therefore, analytical methods that can selectively detect the fluoride ion have been requested in various fields [31]. In this vein, Ahn et al. developed an iminobenzo[*g*]coumarin precursor for fluoride ion sensing (**26**; Figure 6(c)) [32]. The desilylation of silyl enol ether moiety by fluoride ions followed by the intramolecular cyclization produced a compound **24**. In this study, they showed the distribution of fluoride ions in cells and in a live vertebrate, zebrafish, using two-photon microscope (TPM) for the first time. The clear images at deep tissue regions, ∼350 *μ*m depth, represent the superior property of iminobenzo[*g*]coumarin **24** for the two-photon bioimaging.

### 3.2. Imaging of Cell Organs

#### 3.2.1. Mitochondria

**Figure 7:**
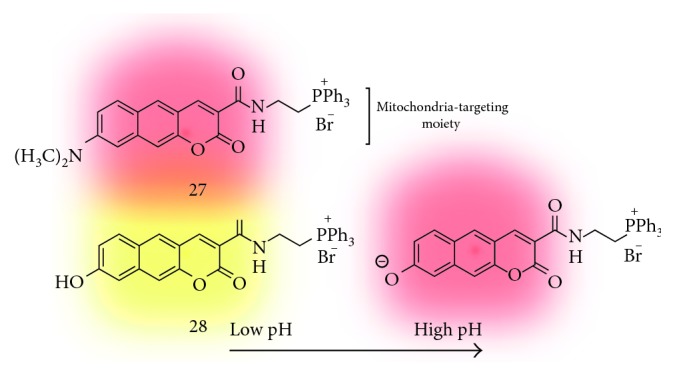
Triphenylphosphonium salt-linked benzo[*g*]coumarin probe for mitochondria tracking (**27**) and measuring mitochondrial pH values (**28**).

Mitochondria is an organelle found in almost all eukaryotic organisms and plays important roles such as production of ATP, protein regulation, storage of calcium ions, and cellular metabolism regulation, among others [33]. Therefore, the defects of mitochondrial function could be directly related to many diseases. So far, various techniques to understand the biological and pathological roles of mitochondria have been developed, and recently, fluorescence methods with imaging materials are used as a standard method to monitor mitochondria dynamics at the subcellular level. In 2014, Kim et al. reported a red-emissive two-photon probe (**27**; Figure 7) based on the benzo[*g*]coumarin for the real-time imaging of mitochondria tracking [17]. The mitochondrial-targeting moiety, triphenylphosphonium (TPP) salt, is linked on the electron-withdrawing part of the benzo[*g*]coumarin core via the amide bond, and the resulting compound **27** exhibited absorption and emission maximum at 470 nm and 626 nm, respectively, with no pH-sensitive changes in the biologically relevant pH range. The staining ability of **27** toward mitochondria was verified by the costaining experiment in the T98G cell line with MitoTracker Green (MTG) as a known mitochondrial labeling marker, and the high Pearson's colocalization coefficient value (0.96) indicates the organ specificity of **27** for mitochondria. In the TPM tissue imaging application using **27**, they observed the evenly distributed mitochondria in the CA1–CA3 region of the rat hippocampal tissue slice at a 200 *μ*m depth.

#### 3.2.2. Mitochondrial pH

The monitoring of pH values and its dynamics in the cellular organs is very important to understand the pH-related biological, physiological, and pathological roles of cells and organisms [34]. In 2016, Kim et al. reported follow-up results that can monitor pH values in the mitochondria using a benzo[*g*]coumarin analogue (**28**; Figure 7) [16]. Probe **28** has a hydroxyl group on the electron-donating position (C-8), and it is protonated or deprotonated at the p*K*_a_ value near 8.0 which is a known pH value of mitochondria. At low pH (pH 4.0), **28** shows absorption and emission maximum at 370 nm and 542 nm, respectively, and the peak is shifted to 453 nm and 604 nm at high pH (pH 10.0) in a ratiometric manner. The high two-photon absorption cross section values (20–70 GM) of **28** at pH 4.0 and 10.0 and the high Pearson's colocalization coefficient (0.95) indicate the ability of selective imaging for mitochondria in the tissue samples. In the cellular imaging, a dense population of mitochondria around the nucleus than in the periphery was observed, and higher mitochondrial pH values in the perinuclear position than in the periphery of cells were also monitored. In a further study, they measured the mitochondrial pH values in the astrocyte from the Parkinson's disease (PD) mouse model and in the rat hippocampal tissue slice. Slightly acidic average pH values in the PD model astrocytes are observed compared with the wild-type astrocytes. The deep tissue imaging results provide average mitochondrial pH values in 7.86–7.88 at CA1, CA3, and the dentate gyrus region.

### 3.3. Sensing of Reactive Species

#### 3.3.1. Nitric Oxide (NO)

**Figure 8:**
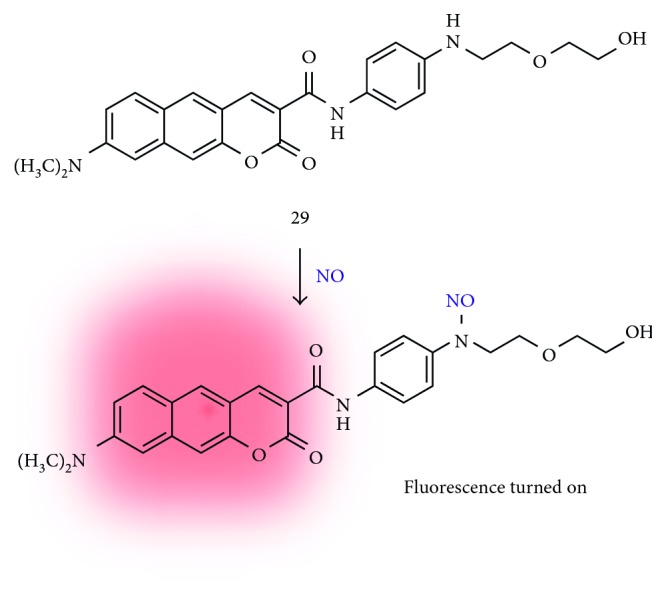
*N*-nitrosation-based benzo[*g*]coumarin probe for nitric oxide (NO) (**29**).

Nitric oxide is a reactive nitrogen radical species, and its functions in living systems have been recognized to be related with cardiovascular, immune, and central nervous systems [35, 36]. So far, various kinds of chemical tools are developed to monitor the location, amount, and retention time in complex microenvironments such as cell and tissue, and these have been applied for the disease study and management. In 2017, Liu et al. reported a new fluorescent probe specifically for NO based on the *N*-nitrosation of the aromatic amine (**29**; Figure 8) [18]. Probe **29** has a benzo[*g*]coumarin backbone with dimethylamine and amide groups at the 3- and 8-position as an electron-donating and electron-withdrawing moiety, respectively. The original fluorescence emission of benzo[*g*]coumarin at 608 nm (at 473 nm excitation) is quenched by the photoinduced electron transfer (PET) from *p*-phenylenediamine moiety and recovered by *N*-nitrosation reaction of NO. The sensitivity toward NO was verified in the screening with the other reactive species such as ClO^−^, H_2_O_2_, ^•^OH, O_2_^−^, NO_2_^−^, and ONOO^−^. The two-photon action cross section values were increased from 2.4 GM to 54 GM at 830 nm under the excitation at 760–900 nm after adding the NO species. The TPM imaging studies for the exogenous and endogenous NO detection were carried out in the live cells (HepG2 cell line), mouse brain tissue, and ischemia/reperfusion injury (IRI) mouse model. Higher fluorescence signals in the TPM images of the IRI model compared with the healthy control represent that NO probe can be applied as a practical tool for studying NO-related biological processes.

#### 3.3.2. Hydrogen Sulfide (H_2_S)

**Figure 9:**
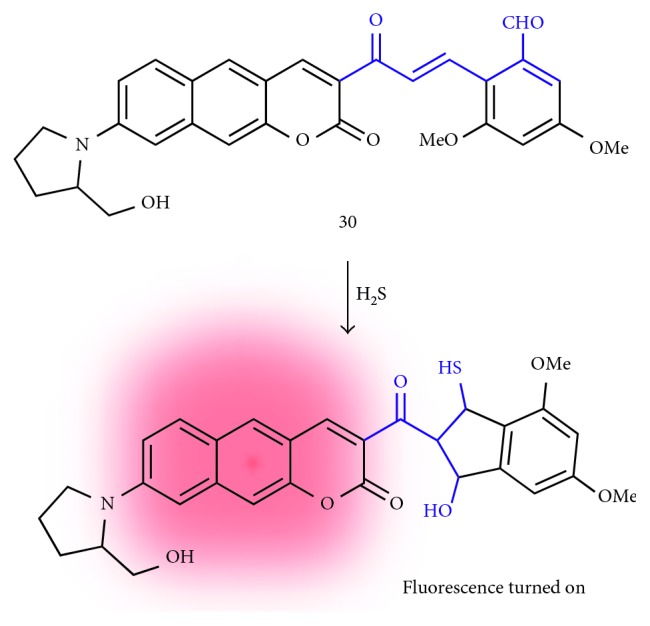
Michael addition and aldol condensation-based fluorescent probe for hydrogen sulfide (H_2_S) (**30**).

Hydrogen sulfide is an endogenous gaseous transmitter along with carbon monoxide (CO) and nitric oxide (NO) [37]. Recent studies of H_2_S revealed that it has a close relationship with neuronal activity, muscle relaxation, insulin management, inflammation, and aging [38]. Very recently, Ahn et al. reported a benzo[*g*]coumarin-based fluorescent probe for monitoring of exogenous and endogenous H_2_S (**30**; Figure 9) [39]. The original fluorescence of the benzo[*g*]coumarin analogue (628 nm fluorescence emission at 485 nm excitation) was enhanced by Michael-type addition followed by aldol condensation of the *α*,*β*-unsaturated carbonyl group with H_2_S [40]. Probe **30** shows the fast response (∼8 min), high selectivity (negligible changes toward biological species), and high sensitivity (detection limit = 0.9 *μ*M) for H_2_S. Bioimaging accessibility for the H_2_S was verified by TPM cellular imaging in the HeLa cell line.

#### 3.3.3. Hypochlorous Acid (HOCl)

**Figure 10:**
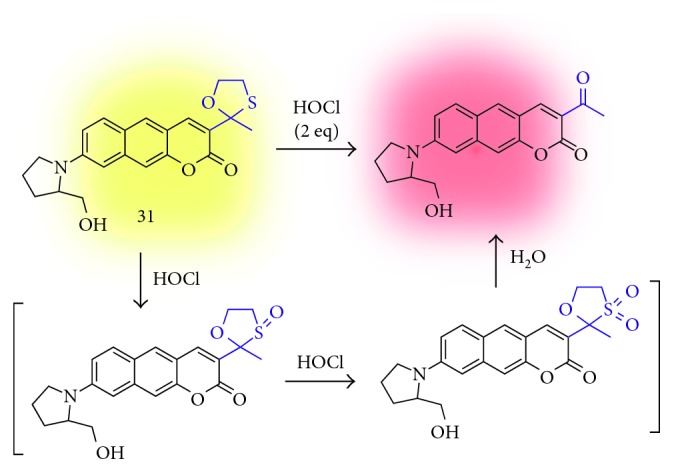
Oxathiolane deprotection-based fluorescent probe for hypochlorous acid (HOCl) (**31**).

Hypochlorous acid is a kind of reactive oxygen species (ROS) [41], and the high level of HOCl is reported in several disorders such as cancer, arthritis, and neurodegenerative disease [42]. Therefore, monitoring the HOCl level and physiological distribution with a pathological mechanism is an important issue. However, the detection of endogenous HOCl is a challenging task due to the low biological concentration, a short lifetime, and a strong oxidizing property [43]. In 2017, Ahn et al. reported a benzo[*g*]coumarin-based ratiometric probe for endogenous HOCl imaging in live cells and tissues (**31**; Figure 10) [21]. An oxathiolane group is substituted at the electron-withdrawing position, and the deprotection into acetyl of this moiety by HOCl causes the intramolecular charge-transfer (ICT) character change of the benzo[*g*]coumarin dye in a ratiometric manner: emission maximum shift from 598 nm (with 424 nm absorption maximum) to 633 nm (with 598 nm absorption maximum). Probe **31** shows a low detection limit at the nanomolar level (34.8 nM) with high sensitivity toward HOCl over various reactive species including H_2_O_2_, ^•^OH, O_2_^−^, ^1^O_2_, and reactive nitrogen species (RNS). The probe **31** and the reaction product give good two-photon action cross section values, 142 GM and 439 GM, respectively. The level of HOCl in the hippocampal slices of the mouse was analyzed by TPM ratiometric imaging with probe **31**, and the slightly higher concentration of HOCl was observed at the dentate gyrus (DG) which is linked to the cognitive ability and memory retention.

### 3.4. Sensing of Disease Biomarkers

#### 3.4.1. Amyloid-Beta Plaque (A*β* Plaque)

Amyloid-beta plaque is an abnormal aggregate of the chemically sticky form of the amyloid-beta peptide (up to 42 or 43 amino acids long) that builds up between nerve cells in the AD patients [44, 45]. Therefore, the extracellular A*β* plaque deposition in the brain is considered as a hallmark of AD. So far, various kinds of contrast agents have been developed for the diagnosis of AD by direct detection of plaques [46]: (i) magnetic resonance imaging (MRI), (ii) positron emission tomography (PET), (iii) single-photon emission computed tomography (SPECT), and (iv) fluorescence imaging. By using the benzo[*g*]coumarin analogue, Ahn et al. found out the selective A*β* plaque staining ability of the iminobenzo[*g*]coumarin analogue (**24**) [47]. In this study, probe **24** selectively stains the A*β* plaques including cerebral amyloid angiopathy (CAA) in the whole brain region successfully. Probe **24** is accumulated in the A*β* plaques accompanying with significant fluorescence increments due to the nature of the donor-acceptor-type dye [48]; strong fluorescence in hydrophobic or viscous environment likes inside of the A*β* plaque. The *in vivo* TPM deep tissue imaging of A*β* plaques in the AD mouse model (5XFAD) treated with probe **24** via intraperitoneal injection shows the high blood-brain barrier (BBB) permeability of **24** and its superior deep tissue imaging ability (∼600 nm depth) with high resolution.

#### 3.4.2. Monoamine Oxidases (MAOs)

**Figure 11:**
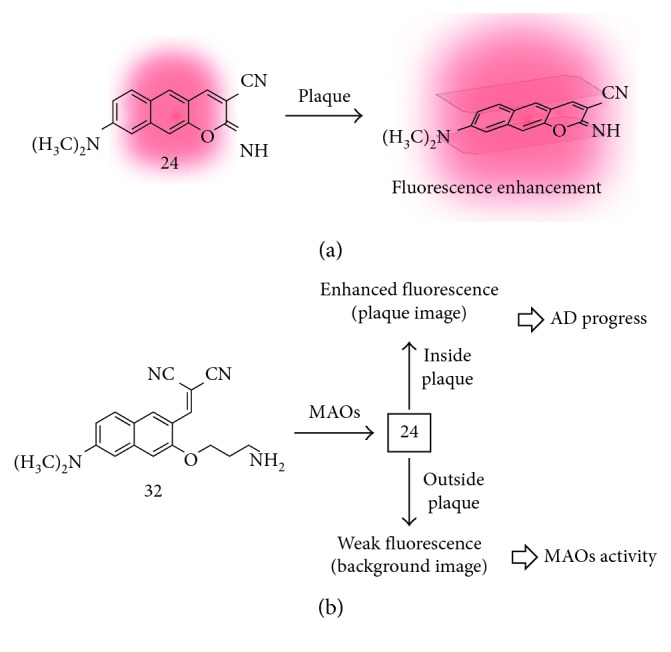
(a) Intercalation-based fluorescent probe for amyloid-beta plaque (**24**). (b) Amine oxidation-based fluorescent probe for monoamine oxidases (MAOs) activity in Alzheimer's disease (AD) (**32**).

**Figure 12:**
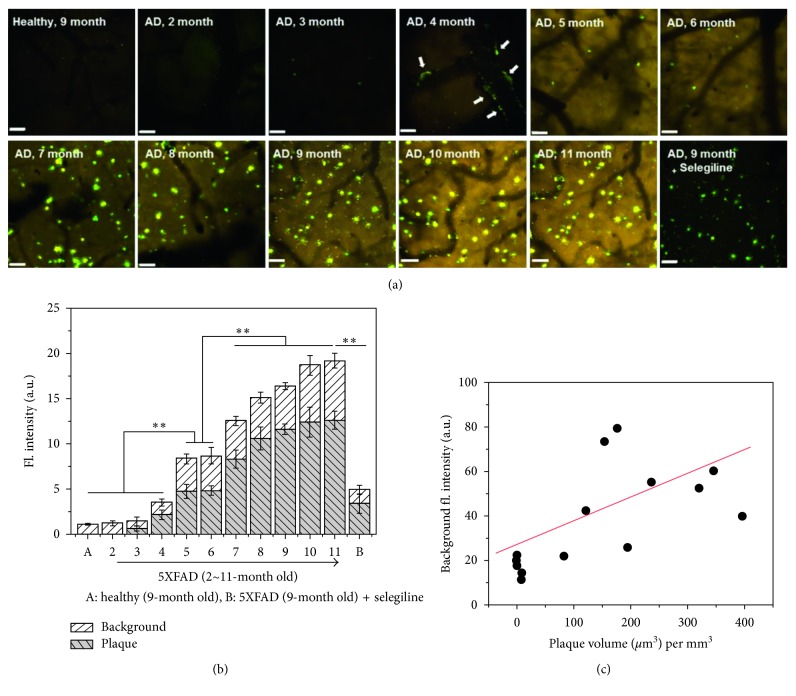
*In vivo* TPM coimaging of MAO activity and A*β* plaques using probe **32**. (a) *In vivo* fluorescence images (from (z)-stack, magnified 20x) of the frontal cortex region of transgenic and healthy mice, obtained after intraperitoneal injection of probe **32**. The scale bar is 60 *μ*m. The images were acquired at 200–300 *μ*m depth from the surface of the cortex. (b) Plots of the average fluorescence intensity of A*β* plaques and background images in (a), respectively. (c) A plot of the background fluorescence intensity versus the plaque volume (*μ*m^3^) per mm^3^. ^*∗∗*^*p*-value < 0.01. Reproduced from [43] with permission from the American Chemical Society, copyright 2016.

Monoamine oxidases are a key enzyme responsible for the regulation of intracellular levels of biogenic amines and amine-based neurotransmitters such as dopamine, adrenaline, and serotonin [49]. A recent study revealed that suppressed or overregulated activity of MAOs is observed in several diseases including cancer and neurodegenerative diseases [50], AD, and Parkinson's disease (PD). In 2012, Ahn et al. developed a fluorescent probe (**32**) that can sense the activity of MAOs by enzymatic cleavage of the aminopropyl moiety followed by intramolecular cyclization and generation of an iminobenzo[*g*]coumarin (**24**) (Figure 11) [19]. In the intensive study, they applied probe **32** to find a correlation between activity of MAOs and AD progress in the animal model by using TPM [47]. Interestingly, significant background signal enhancement that correlated with MAO's activity was observed in older AD mice. The MAO's enzymatic product **24** is accumulated from the outside of A*β* plaques to the inside, and the fluorescence intensity is increased as growing older (increased numbers and size of A*β* plaques in the brain of the mouse model) (Figure 12). This is the first demonstration for following activity of MAOs and AD progress *in vivo*.

### 3.5. Deep Tissue Imaging

The fluorescence tissue imaging has emerged as the strong tool for studying biological events and clinical applications. In particular, the fluorescence deep tissue imaging with high resolution offers collective information of the cellular processes in a macroscopic view. Among the various imaging techniques, TPM has shown superior performance for deep tissue imaging. However, a key limitation for TPM-based deep tissue imaging is the autofluorescence interference from intrinsic biomolecules in the tissue such as nicotinamide adenine dinucleotide (NADH) and its phosphate analogue (NADPH), riboflavin, and flavoproteins [22]. The autofluorescence issue when using the known two-photon absorbing dyes has been solved by technical methods such as tuning the excitation wavelength, reducing the laser power, and changing the detection channel and/or sensitivity.

**Figure 13:**
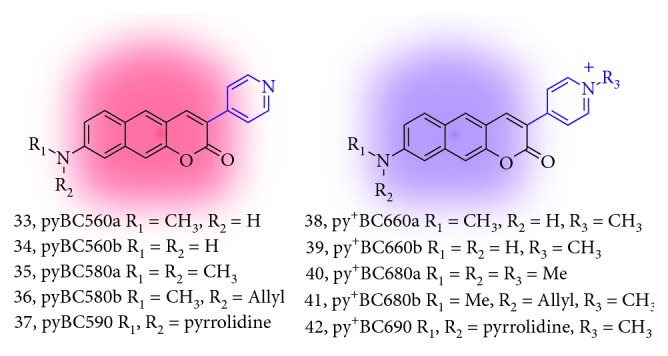
Red/far-red-emitting pyridinium-benzo[*g*]coumarin derivatives (**33**–**42**).

To overcome this issue, Ahn et al. focused on the systematic study of the new two-photon absorbing dyes based on benzo[*g*]coumarin analogues. In 2017, they reported pyridyl/pyridinium-benzo[*g*]coumarin analogues which have far-red-emitting (585–691 nm) fluorescence (**33**–**42**; Figure 13; Table 2) [22]. They optimized the wavelength of benzo[*g*]coumarin analogues that can address the autofluorescence issue. The pyridinium group at electron-withdrawing position (C-3) makes the significant wavelength shift to the far-red region (660–691 nm) from the parent benzo[*g*]coumarin or pyridyl-benzo[*g*]coumarin. In the brain tissue imaging with Py + BC690 (**42**), the clear deep tissue TPM imaging after an optical clearing process (BABB clearing) [51] was observed at the stage down to 1380 nm depth. The imaging depth indicated the high tissue uptake of dye and penetration ability of **42** which are important features as a bioimaging agent.

## 4. Summary and Outlook

Since the first report about the pi-extended structure of coumarin, the tremendous knowledge and experimental results have been accumulated. In this focused review, the basic photophysical property, synthetic method, and applications of benzo[*g*]coumarin analogues are summarized. Molecular structures of linearly pi-extended benzo[*g*]coumarin analogues are expected to provide a longer excitation and emission wavelength at the red/near-infrared region with larger two-photon absorbing ability, and the experiment results have given evidences. In addition, their rigid conformation with facile function granting serves the high quantum yield, superior photostability/chemical stability, and applicability for the development of molecular probes. Some of the benzo[*g*]coumarin analogues showed promising two-photon absorbing properties holding great promise in the development of two-photon bioimaging probes to sense biologically important species. Most of the bioimaging applications of benzo[*g*]coumarin analogues are carried out very recently; therefore, we hope that this review inspires scientists to develop more advanced systems with useful practical applications such as disease biomarker sensing for prognosis and diagnosis.

## Figures and Tables

**Table 1:** Photophysical properties of the benzo[*g*]coumarin derivatives **11**–**21**. The wavelengths are derived from the highest intensity values in the absorption and fluorescence emission spectra in the described solvent.

Compound	*λ*_abs_ (nm)	*λ*_emi_ (nm)	Solvent	Reference
**1**	320	459	CH_3_CN	[13]
**2**	332	534	CH_3_CN	[13]
**3**	337	549	CH_3_CN	[13]
**4**	345	547	CH_3_CN	[13]
**5**	334	515	CH_3_CN	[13]
**6**	326	510	CH_3_CN	[13]
**7**	335	533	CH_3_CN	[13]
**8**	357	466	CHCl_3_	[14]
**9**	450	603	DI H_2_O	[12]
**10**	355	539	DI H_2_O	[12]
**11**	467	623	EtOH	[15]
**12**	413	599	DI H_2_O	[12]
**13**	370/453	542/604	pH 4/pH 7 buffer	[16]
**14**	470	626	pH 7.4 buffer	[17]
**15**	450	600	pH 7.4 buffer	[18]
**16**	357	522	DI H_2_O	[12]
**17**	435	582	DI H_2_O	[12]
**18**	444	607	EtOH	[15, 19]
**19**	431	591	CH_3_CN	[20]
**20**	487	633	pH 7.4 buffer	[21]
**21**	527	691	EtOH	[22]

**Table 2:** Photophysical properties of pyridinium-benzo[*g*]coumarin derivatives (**33**–**42**).

Compound	*λ*_abs_ (nm)	*λ*_em__i_ (nm)	Brightness	GM
**33**	n.r.	n.r.	n.r.	n.r.
**34**	n.r.	n.r.	n.r.	n.r.
**35**	445	585	n.r.	n.r.
**36**	n.r.	n.r.	n.r.	n.r.
**37**	n.r.	n.r.	n.r.	n.r.
**38**	506	663	749	n.d.
**39**	499	660	510	n.d.
**40**	513	681	2089	n.d.
**41**	511	680	1173	150
**42**	527	691	799	160

The wavelengths, brightness, and GM values are derived from the highest intensity values in the absorption and fluorescence emission spectra. Brightness: molar extinction coefficient (LMol^−1^·cm^−1^) × quantum yield (Φ_F_); GM: two-photon absorption cross section (TPACS, Goeppert-Mayer unit); n.r.: not reported; n.d.: not determined.
